# Inteins: Localized Distribution, Gene Regulation, and Protein Engineering for Biological Applications

**DOI:** 10.3390/microorganisms6010019

**Published:** 2018-02-28

**Authors:** Theetha L. Pavankumar

**Affiliations:** Department of Microbiology and Molecular Genetics, Briggs Hall, One Shields Ave, University of California, Davis, CA 95616, USA; pavan@ucdavis.edu; Tel.: +1-530-754-9702; Fax: +1-530-754-8973

**Keywords:** inteins, splicing, Hint domains, DNA replication, repair and recombination, protein engineering

## Abstract

Inteins are self-splicing polypeptides with an ability to excise themselves from flanking host protein regions with remarkable precision; in the process, they ligate flanked host protein fragments. Inteins are distributed sporadically across all three domains of life (bacteria, archaea, and unicellular eukaryotes). However, their apparent localized distribution in DNA replication, repair, and recombination proteins (the 3Rs), particularly in bacteria and archaea, is enigmatic. Our understanding of the localized distribution of inteins in the 3Rs, and their possible regulatory role in such distribution, is still only partial. Nevertheless, understanding the chemistry of post-translational self-splicing of inteins has opened up opportunities for protein chemists to modify, manipulate, and bioengineer proteins. Protein-splicing technology is adapted to a wide range of applications, starting with untagged protein purification, site-specific protein labeling, protein biotinylation, isotope incorporation, peptide cyclization, as an antimicrobial target, and so on. This review is focused on the chemistry of splicing; the localized distribution of inteins, particularly in the 3Rs and their possible role in regulating host protein function; and finally, the use of protein-splicing technology in various protein engineering applications.

## 1. Introduction 

Inteins are intervening polypeptides with an ability to splice themselves out from the flanking protein fragments (exteins) post-translationally. Inteins (intervening proteins) were first found associated with the *VMA1* gene (also known as *TFP1*) that encodes for α subunit of vacuolar membrane H^+^-translocating adenosine triphosphatase (H^+^-ATPase) of *Saccharomyces cerevisiae* [1]. The TFP1 gene product was observed to be larger (118.6 kDa) than the estimated 69 kDa, with N- and C-terminal regions being homologous to the similar H^+^-ATPases [1]. Later, work by Kane et al. convincingly demonstrated that the TFP1 precursor protein (118.6 kDa) splices itself out post-translationally into 69-kDa (H^+^-ATPase) and 50-kDa proteins [2]. Since then, several hundreds of inteins have been reported [3].

Inteins are widely dispersed in nature. Their broad phylogenic distribution across all three domains of life suggests that inteins have ancient origins. Despite their wide distribution, they are absent in multicellular organisms. Inteins are somehow analogous to introns. The splicing of inteins can occur either spontaneously or under favorable conditions. In most cases, inteins are expressed as a single contiguous polypeptide (*cis*-splicing inteins); in some instances, they are transcribed and translated separately as split or *trans*-splicing inteins. These trans-splicing inteins re-associate via the zipper-like interface and perform a splicing reaction [4,5,6]. Both contiguous and split intein splicing processes and their chemistry of making and breaking peptide bonds have been exploited to develop various protein engineering techniques. 

## 2. Hint Domain Superfamily and Different Forms

Hint (Hedge-Hog/Intein) is a protein domain containing fundamental characteristic features needed for the protein-splicing reaction to occur. Hind domains exist in different forms. They are 140 to 160 amino acid long polypeptides containing 4–6 conserved motifs. They are primarily comprised of three β-sheets and two α-helices linked through loop regions. Hint domain was shown to fold into a horseshoe-like core structure with a pseudo-two-fold symmetry [7]. The proper folding of the Hint protein domain is all that seems to be an essential criterion for the precise protein splicing process to happen, and thus, Hint domains are the key players in the protein-splicing process. The Hint domain superfamily is comprised of three important subfamilies: (1) Intein-Hint domain, (2) Hedgehog-Hint domain, and (3) Bacterial intein-like (BIL)-Hint domains. These subfamilies are largely varied by the type of functional domains associated with them and the way they splice-out. 

## 3. Intein-Hint Domain 

**Figure 1:**
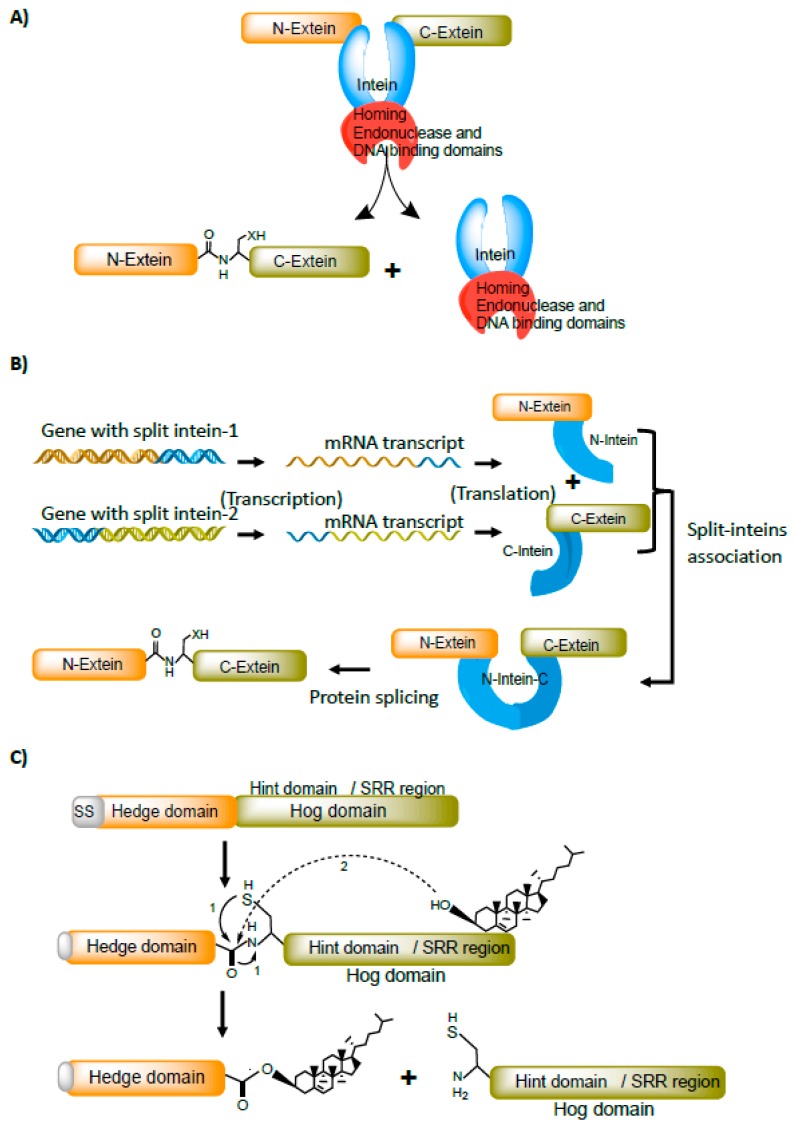
Different forms of the hint domain. (**A**) Inteins containing homing endonuclease (HE) and DNA binding domains. Inteins with HED are selfish genetic mobile elements capable of integrating into the alleles. (**B**) Split inteins are *trans-*splicing elements that are transcribed and translated separately and have the ability to associate *in-trans*, and splice out. (**C**) Hedgehog proteins with hint domain (hog domain). The conserved glycine and cysteine residues rearrange to form a thioester bond (step-1; N to S acyl rearrangement). The hydroxyl group of cholesterol attacks thioester bond (step-2; *trans*-esterification) and links cholesterol to the hedge domain releasing hog domain. SS, a processed signal-sequence peptide at the N-terminus of the Hedge domain.

Inteins are part of the Hint domain superfamily. All inteins have a protein-splicing Hint domain. However, inteins vary widely by having different functional domains embedded within them. Many inteins have homing endonuclease (HED) and DNA binding domains embedded within them. Inteins with HED domain splice out spontaneously from the precursor protein by ligating N-terminus extein (N-extein) with C-terminus extein (C-extein) to form a functional protein (Figure 1A). Inteins embedded with HED and DNA binding domains are selfish genetic mobile elements. They recognize a nucleotide sequence ranging from 14–40 bp and induce sequence-specific double-strand breaks (DSBs) on an intein/HED free allele [8]. The intein/HED domain is then mobilized into an intein/HED free allele by DSB-mediated DNA repair via homologous recombination. However, many inteins have apparently lost the HED and DNA binding domains during evolution. Inteins with or without HED and DNA binding domains are contiguous and are produced in a single polypeptide form. 

Unlike contiguous inteins, certain inteins are produced in two or more polypeptide parts. These split inteins are transcribed and translated separately. Although split inteins are being produced separately, they retain all the hint domain features needed for the splicing process [4,9,10]. They associate in-trans and perform a splicing reaction (Figure 1B). Split inteins are naturally found in cyanobacteria [9,11]. Intriguingly, these are inserted in a conserved motif of essential genes, particularly, in DNA replication machinery [9]. It is speculated that split inteins resulted from genomic arrangements that split the contiguous intein into two functionally independent entities during evolution. The proper Hint domain folding is the essential parameter for splicing to occur in both contiguous and split inteins.

## 4. Hedgehog-Hint Domain 

Hedgehog signaling proteins are the key regulators of the developmental-signaling pathway in metazoans. Hedgehog proteins are composed of three domains. The N-terminal region of the protein is the Hedge domain and, the C-terminal region (Hog) is comprised of a Hint domain and a sterol recognition region (SRR). The C-terminal region with Hint domain has the same structural fold as inteins [7] and is responsible for the maturation of the N-terminus Hedge protein. During the maturation process, the conserved glycine and cysteine residues of Hog domain are rearranged to form a thioester. The hydroxyl-oxygen of the cholesterol molecule attached to the SRR region attacks the thioester bond. During the attack, the cholesterol molecule gets transferred on to the Hedge protein via ester linkage and the modified N-terminus region (Hedge domain), with cholesterol at C-terminus splice-out from the C-terminal Hog-hint region [12] (Figure 1C).

## 5. Bacterial-Intein-Like Hint Domain

**Figure 2:**
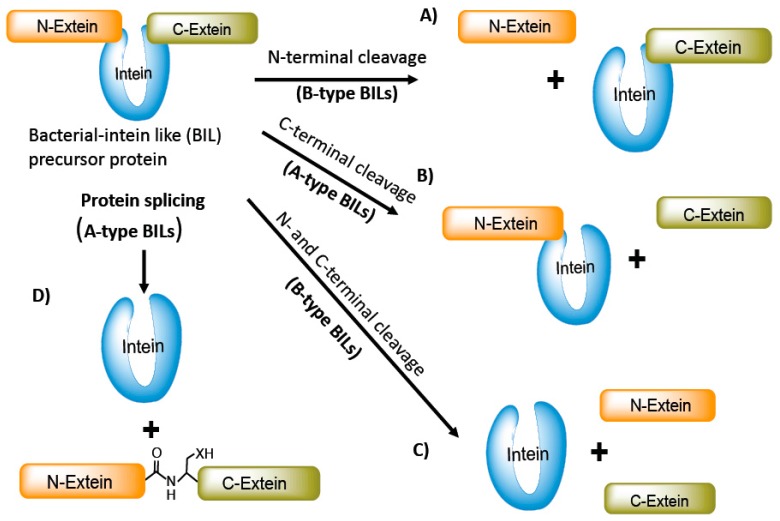
Bacterial intein-like (BIL) proteins and their splicing behavior. BILs precursor proteins follow the non-canonical splicing mechanism. A-type BILs often lack nucleophilic residue at the C-terminal flanking region and are susceptible to C-terminal cleavage. B-type BILs undergo both C-terminal and/or N-terminal cleavage (**A**,**C**). A-type can splice completely to produce fully functional protein and often undergo C-terminal cleavage (**B**,**D**).

Bacterial-intein-like (BIL) domains are similar to intein and Hedgehog-hint domain family proteins but differ in certain sequence features [13]. The phylogenic distribution and type of host protein in which the BILs are inserted also vary compared to the intein and Hog-Hint domain family. Unlike inteins, BIL domains are inserted in non-conserved variable regions of proteins of divergent bacteria. Two different types of BIL domains have been identified: A- and B-type. Both A- and B-type domains can self-cleave at the N- or C-terminus of the BIL domains. The A-type BIL domains, in some instances, follow an alternative splicing mechanism, whereas B-type domains appear to follow the canonical protein-splicing mechanism [14] (Figure 2). 

## 6. Mechanism of Intein-Mediated Protein Splicing

**Figure 3:**
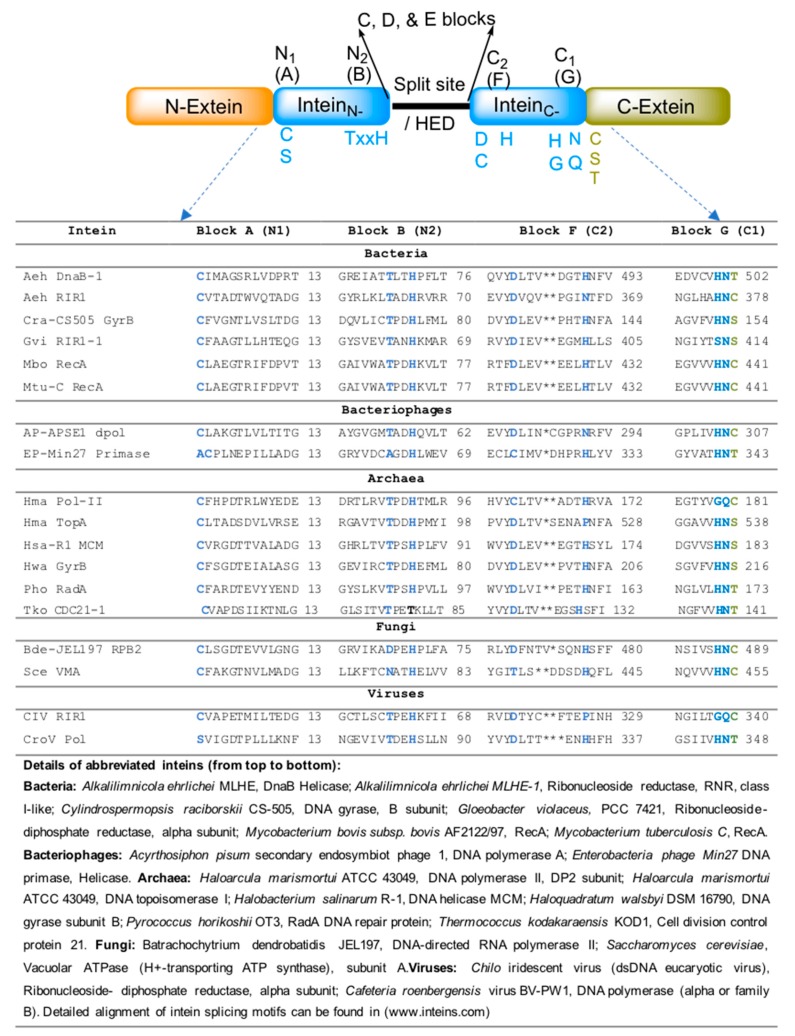
Important motifs and conserved splicing elements of inteins. Inteins contain four important motifs: The N-terminal region with N_1_ and N_2_; and the C-terminal region with C_1_ and C_2_ motifs. The important splicing amino acid residues of each motif are indicated. The conserved amino acid residues (C, S, and T) on the C-extein are also indicated. These amino acids are also important for the splicing process to occur. A list of selected intein-associated proteins of bacteria, bacteriophages, viruses, and fungi, and their protein sequence alignment of conserved A, B, E, and F motifs, is also given.

Protein splicing is all about breaking a peptide bond (at intein–extein junctions) and forming a new peptide bond between extein polypeptides, but in a meticulous way. It is a precise chemical reaction that occurs between specific conserved amino acids residing within inteins and exteins. Protein splicing is typically a single-turnover reaction and, in general, does not require cofactors. Sequence alignment of inteins obtained from intein database (www.inteins.com) revealed that inteins have four important splicing motifs, each comprising highly conserved similar amino acids. These motifs are further designated into seven blocks: A to G. Among the seven blocks, A, B, F, and G (also known as N1, N2, C2, and C1, respectively) are the essential blocks and reside within the intein region (Figure 3). 

**Figure 4:**
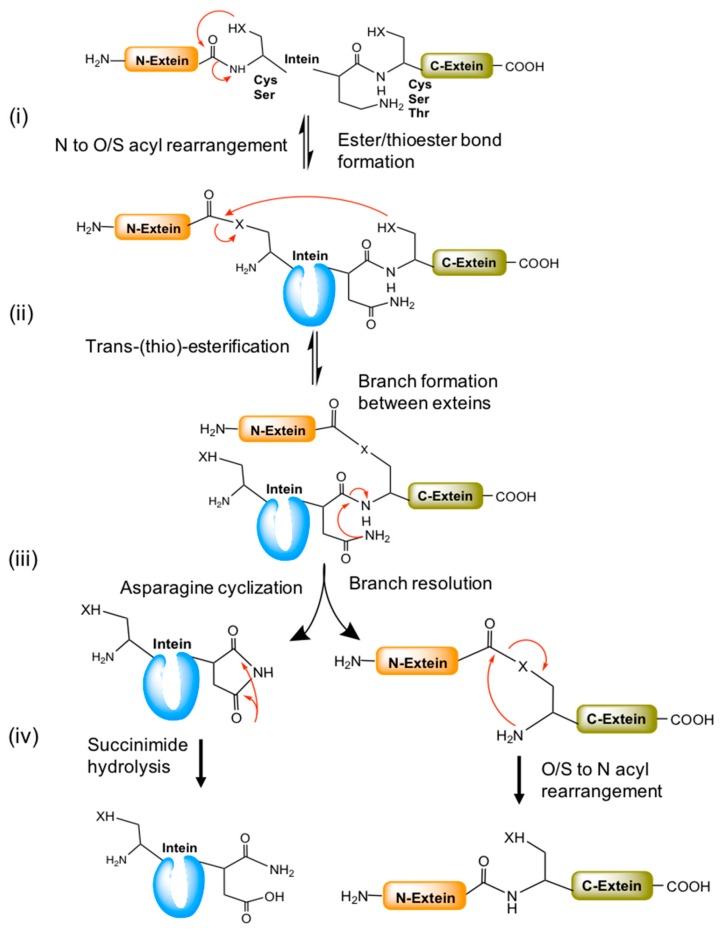
A general mechanism of the canonical splicing pathway. (**i**) Splicing begins with ester/thio-esterification (serine or cysteine residues) by N to O/S acyl rearrangement at the N-extein end. (**ii**) The esterified N-extein is linked to the C-extein by *trans*-(thio) esterification (by C, S or T). The branch resolution and Asn cyclization occurs in the next step with the help of conserved penultimate histidine residues. (**iii**) Later, the N- and C-exteins undergo O/S to N acyl rearrangement to form a new peptide bond between them. (**iv**) The cyclized asparagine converted into succinimide by hydrolysis. Red arrows represent the chemical reaction/s between the functional groups indicated.

Splicing mechanisms of different classes of inteins have been investigated in detail. However, it is hard to define a universal mechanism by which the protein-splicing reaction works. In some instance, it detours from canonical splicing pathway as some conserved functionally similar amino acids need assistance from the adjacent amino acids to complete the splicing reaction. A generalized mechanism of canonical splicing involves the following steps;

1. Ester/thioester bond formation by N to O/S acyl rearrangement,
2. Trans-(thio)-esterification between exteins,
3. Asparagine cyclization, and
4. Spontaneous hydrolysis of the amino-succinamide residue and O/S to N acyl rearrangement to form a peptide bond between exteins (Figure 4).

The splicing process can occasionally deviate from canonical splicing as a consequence of variation in the conserved amino acids of the N1, N2, C2, and C1 blocks (Blocks A, B, F, and G, respectively) (Figure 3). The lack of block-N2 or penultimate histidine in block-C1 affects the splicing process. For example, the *Thermococcus kodakaraensis* CDC21-1 (TkoCDC-21-1) intein has threonine instead of histidine at block-N2 [15]. In this case, a lysine residue (K58) residing outside of the standard intein conserved motifs seems to catalyze the initial N-S acylation reaction [15]. Similarly, the DnaE inteins of cyanobacteria have either serine or alanine in place of histidine at block-C1 [9]. These histidine residues are important for initiating N to O/S acyl rearrangement and asparagine cyclization, respectively [15,16]. In addition, an external nucleophilic attack (such as water and thiol) on the (thio) ester intermediate can cause N-terminal cleavage. Similarly, C-terminal cleavage could also occur if asparagine cyclization occurs during the delay or absence of transesterification. 

## 7. Localized Distribution of Inteins and Post-Translational Regulation 

Inteins are distributed sporadically across all three domains of life. About 24%, 47%, and 1% of the total genome of bacteria, archaea, and lower eukaryotes, respectively, contain inteins [17]. Intriguingly, 62% and 67% of inteins of bacteria and archaea reside in DNA replication, recombination, and repair proteins. In particular, inteins are embedded in DNA polymerases, DNA topoisomerases, DNA helicases, DNA strand exchange proteins, and ribonucleotide reductases. Inteins are often found inserted at critical functional sites such as the phosphate-binding loop (P-loop) or the catalytic or ligand binding sites of many essential DNA replication and repair proteins [18]. Also, the insertion site is varied across the host proteins. DNA helicases (such as DnaB, PcrA, and UvrD) and DNA strand-exchange proteins (such as RecA and RadA) have inteins inserted at the P-loop, and DNA polymerases and topoisomerases have found intein insertion at catalytic or ligand binding sites [18,19,20]. Inteins are also found in the ribonucleotide reductase large subunit of the *Chilo* iridescent virus [21]. A list of proteins with intein insertion is given in Table 1.

The discovery of enigmatic distribution and preferred insertion site of inteins has left scientists with more questions than answers.

1. Why do inteins localize in DNA replication, recombination, and repair proteins, and in RNA transcription machinery?
2. Is there any significance of intein association with particular conserved regions (like P-loop of NTPases or catalytic sites) of essential proteins?
3. Do they have a regulatory role in DNA replication machinery under stress conditions? or
4. Is it just a selective pressure that retains intein association with particular regions of certain functional proteins, as argued earlier [17]?

Still, the localized distribution and preferred insertion of intein into essential genes is still a matter of debate. However, recent advances in intein biology indicate that inteins may act as environmental cues. The pathogenic bacteria such as *Mycobacterium tuberculosis* and *Mycobacterium leprae* have intein inserted in r*ecA* gene [22]. Inteins inserted into *M. tuberculosis* and *M. leprae* are different in size, sequence, and location of insertion. Importantly, the *in-vivo* splicing of both inteins also varies. *M. tuberculosis* RecA precursor protein splice out spontaneously when expressed in *E. coli* and also, when N- and C-terminal purified fragments are provided *in-trans* in the presence of DTT [23]. In contrast, *M. leprae* RecA precursor protein could splice out only in native cells and in *M. smegmatis,* not in *E. coli* [22,24]. This observation suggests that the splicing of *M. leprae* RecA precursor protein is a host-specific reaction and requires an unknown splicing cofactor specific to *M. leprae* and *M. smegmatis*. 

There is growing evidence of the existence of conditional protein splicing, in which the splicing process is regulated by many factors such as redox state, temperature, pH, and DNA. It is observed that some inteins contain two cysteines at the active splicing site and have the potential to form a disulfide bridge. In the case of the *Pyrococcus abyssi* DNA polymerase II intein precursor, these two cysteine residues form an intramolecular disulfide bond that inhibits protein splicing [25]. It is speculated that *P. abyssi* is anaerobic and the presence of oxygen may pose oxidative stress. The oxidative stress may promote disulfide bridge formation and thus inhibit DNA polymerase II precursor splicing during oxidative stress. Similarly, the SufB intein of *M. tuberculosis* is found to act as a sensor for oxidative and nitrosative stress [26].

Protein splicing is also regulated by temperature. An intein inserted at the ATPase domain of RadA (a DNA strand-exchange protein) of the hyperthermophilic archaeon *Pyrococcus horikoshii* seems to be regulated in a temperature-dependent manner. It is demonstrated that efficient splicing occurs only at temperatures between 65 to 85 °C and is controlled by native exteins (the host RadA protein fragments) [27]. *P. horikoshii*, being a hyperthermophilic archaeon, temperature-dependent modulation of RadA production, may serve as a regulatory mechanism in this archaeon.

Deoxyribonucleic acids (DNA) are the essential genetic elements of living organisms. A recent study indicates that ssDNA (single-stranded DNA) and DNA damage response both seem to trigger the protein splicing of the *P. horikoshii* RadA protein. Interestingly, the stimulation is specific to ssDNA but insensitive to dsDNA (double-stranded DNA), dNTPs, and RNA [28]. All the evidence given above points to the post-translational regulation of a protein’s function through splicing. Therefore, it can be envisaged that protein splicing is a post-translational regulatory mechanism, by which biological functions are regulated to allow the individual to thrive under various stress conditions.

## 8. Applications of Intein Splicing in Protein Engineering and Biological Applications

The intein-mediated way of breaking and making a peptide bond offers promising scope for protein engineering. Since the discovery of inteins and their splicing chemistry, protein chemists have been striving to adapt and develop a wide variety of bioengineering techniques for biological applications. To date, the splicing technique has been successfully employed in protein purification, protein modifications, peptide cyclization, and as intein-based biosensor and reporter systems. Inteins are also being investigated as a potential target for antimicrobial drugs.

## 9. Inteins in Protein Purification

**Figure 5:**
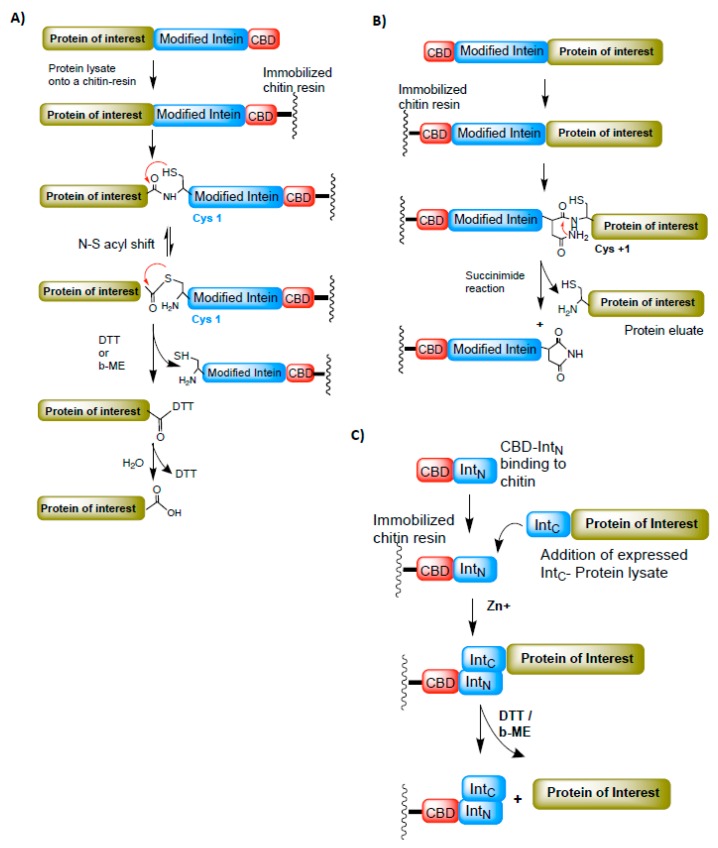
The intein-based protein purification techniques. (**A**) A method to purify the protein of interest (POI). The C-terminus of POI is fused to the N-terminus intein-CBD (chitin-binding domain). The POI-Intein-CBD fusion is expressed and immobilized onto the chitin resin. The POI is later cleaved from the bound intein-CBD-chitin resin by adding DTT or β-ME. (**B**) A modified intein-based purification method to purify N-terminus-fused POI attached to the C-terminus of intein-CBD domain. In this case, the POI is cleaved from the fused domain by succinimide hydrolysis. (**C**) Split intein-based purification system. The N-terminus intein (int_N_) fused to CBD is immobilized onto the chitin resin and the C-terminus intein (int_C_) fused to POI is allowed to trans-associate with int_C_-CBD domain. Zinc is added to minimize the splicing reaction during the binding process. Later, POI is cleaved off from the bound fractions by adding DTT or β-ME. Red arrows represent the chemical reaction/s between the functional groups indicated.

Affinity chromatography is a widely adopted technique for protein purification. It requires affinity tags such as 6XHis, FLAG octapeptide, Glutathione S-transferase (GST), Maltose-binding protein (MBP), or Chitin-binding protein (CBP) to be attached either at the N- or C-terminus of the protein of interest. Often, these tags interfere with a protein’s function and need to be removed by proteases (such as precision protease, Thrombin, or Factor Xa). In some instances, affinity-tag removal by proteases is time-consuming and may leave additional amino acids on the protein of interest. The discovery of intein-mediated protein splicing process enabled the purification of recombinant proteins without affinity tags (fused affinity tags are cleaved from the protein of interest during intein-mediated splicing) and with no additional amino acids on them, as demonstrated by Chang et al. in 1997 [29]. In this study, a modified intein from *Saccharomyces cerevisiae* (*Sce* VMA intein) was fused to the chitin-binding domain (CBD) from *Bacillus circulans* as an affinity tag. In general, the protein of interest is cloned at the N-terminus of the intein-CBD fusion domain and immobilized on the chitin column (Figure 5A). The splicing (cleavage) is induced by adding either DTT or β-ME. This technique was further extended to fuse the N-terminus of the protein of interest with the C-terminus intein and purify the proteins by the C-terminus splicing reaction [30] (Figure 5B). However, in this case, cysteine (Cys^+1^) on the protein of interest (Extein) is required for the splicing process to occur efficiently. 

Similar to the Sce-VMA intein-mediated purification system, a *Mycobacterium xenopi* Gyrase-A intein containing a purification technique was also developed. Here temperature is used as a splicing factor to cleave an immobilized target protein [31]. Nevertheless, preventing or minimizing the cleavage of protein precursor during expression and rapid cleavage of protein of interest during purification are the essential parameters for a successful intein-mediated purification system. Since then, many investigators have tested and developed efficient purification systems using modified inteins fused to different affinity tags [32,33,34,35,36]. 

The split intein chemistry also contributed remarkably to the protein purification system. Their ability to associate *in-trans* and splice out rapidly have added a next level to the purification of recombinant proteins. Many engineered split inteins are employed to develop better and more efficient purification systems [4,10,37,38,39,40,41,42]. One such purification system developed using an engineered split intein from *Nostoc punctiforme* DnaE is found to be efficient and robust in cleaving the protein of interest at a remarkably faster rate [4,43,44]. The steps involved in protein purification using *N. punctiforme* DnaE split inteins are illustrated in Figure 5C. 

## 10. Protein Modifications Using Splicing Chemistry

**Figure 6:**
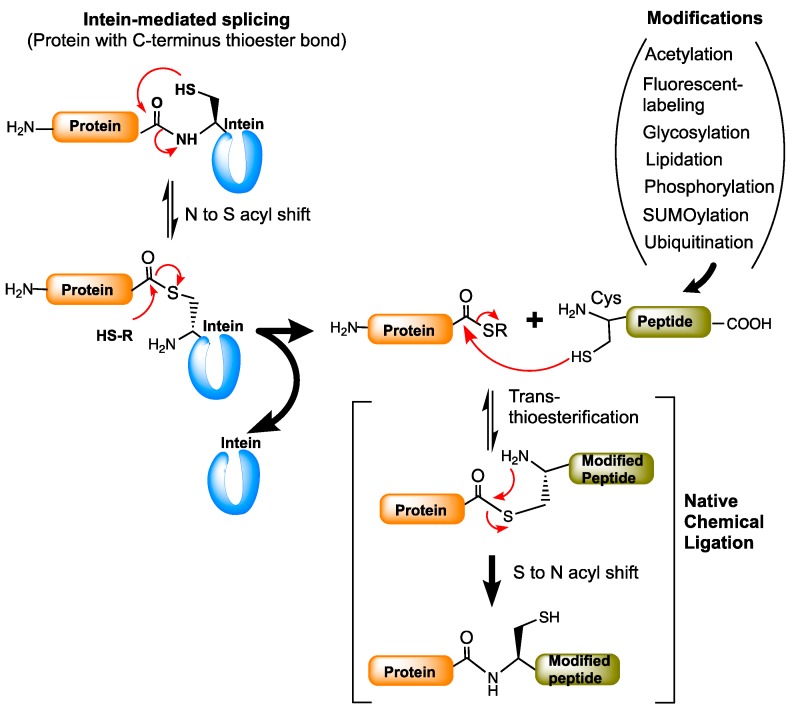
Protein modifications by expressed protein ligation (EPL). The protein of interest fused to the intein is expressed and purified in esterified form. The esterified protein is then attached to the modified peptides (or proteins) by trans-(thio)esterification process using native chemical ligation (NCL). Red arrows represent the chemical reaction/s between the functional groups indicated.

The chemistry of ligating two peptides goes back to the 1990s. A Native Chemical Ligation (NCL) method developed by Kent and co-workers has been found to be tremendously useful in peptide chemistry [45]. It is a reaction that ligates two peptides by allowing a reaction between a peptide with C-terminal thioester (α-thioester) and a peptide with a cysteine at the N-terminus end. NCL is a technically challenging method and is limited to producing polypeptides up to 15 kDa. The limitations of NCL were eliminated by intein-mediated protein splicing chemistry-based methods such as expressed protein ligation (EPL) and protein trans-splicing (PTS). Expressed protein ligation [46,47] is essentially similar to NCL but involves an intein-based splicing reaction to produce the protein of interest with a C-terminal thioester to which cysteine-containing chemically modified proteins or peptides are ligated under mild aqueous conditions (Figure 6). The protein trans-synthesis [48] is very similar to the split inteins splicing process (Figure 1B), but in the case of PTS the N- and C-exteins are the proteins of interest with modifications. Both EPL and PTS are used for linking two proteins of interest with desired modifications for biological applications. Both techniques have proven useful in post-translational modifications (PTMs) of proteins. 

Expressed protein ligation is the most common method used for the semisynthesis of post-translationally modified proteins. EPL has been used for protein modifications such as acetylation, phosphorylation, ubiquitination, SUMOylation, glycosylation, lipidation, and in the fluorescent labeling of proteins [49]. EPL is widely used in the phosphorylation of protein kinase Csk [47] to study the structural basis of heteromeric Smad protein [50,51], in the preparation of glycoproteins [52], for ubiquitination of PCNA (proliferating cell nuclear antigen) [53], and in histone proteins [54]. It is also employed in site-specific labeling of proteins for the Förster resonance energy transfer (FRET) studies [55]. More detailed biological applications of intein-based EPL and PTS methods are reviewed in [49,56].

Cyclized proteins or peptides are found naturally in bacteria, plants, and mammals. Cyclization is a process of linking the N-terminus of a peptide (or protein) with its C-terminus end by a peptide bond. Cyclosporine is one such cyclized peptide found in fungi and is being used as an immunosuppressant in the medical field. Cyclization of proteins or peptide seems to offer stability, improved biological activity, and affinity compared to their counterparts. Many naturally existing cyclotides (cyclized peptides) have antimicrobial, anticancer, and anthelmintic properties. The cyclization of a target protein can be carried out by sandwiching the target protein between the N- and C-inteins, as shown by using a split intein of the *dnaE* gene from *Synechocystis* species PCC6803 [57]. Polypeptide cyclization is shown to produce biologically active, fast-folding, and denaturation-resistant recombinant proteins compared to their counterparts [58]. Hence, cyclization of synthetic peptides is a subject of interest in pharmaceutical industries. Peptide cyclization of c-Crk, β-lactamase, and green fluorescent protein (GFP) was performed to obtain better derivatives [59,60,61]. Detailed information on protein cyclization and applications of both EPL and PTS in peptide cyclization is given in [62].

Intein- and split-intein-mediated splicing chemistry is further extended to in vivo applications such as developing an intein-based biosensor (redox-state sensor in *E. coli*), in sensing DNA methylation, to understand site-specific proteolysis and study protein–protein interactions [49,56,63]. 

## 11. Inteins as Microbial Drug Target

Many inteins reside in pathogenic bacteria, such as *Mycobacterium tuberculosis*, *Mycobacterium leprae,* and *Coxiella burnetti*. Inteins are also found in pathogenic fungi like *C. neoformans*, *C. gattii*, and *Histoplasma capsulatum*. As inteins are largely found in essential genes of many organisms including *M. tuberculosis* and *M. leprae,* inhibiting intein splicing may offer a target for antimicrobial drugs. Cisplatin, an anticancer drug, has recently been shown to inhibit the protein-splicing process in mycobacteria [64]. Nevertheless, cisplatin cannot be used as an antimicrobial agent as it is being used as an anticancer drug and may pose severe side effects if used as an antimicrobial agent. At the least, intein splicing inhibition offers a novel microbial drug target, particularly for the treatment of tuberculosis and leprosy.

## 12. Conclusions

The localized distribution of inteins, particularly in DNA replication and repair proteins at the critical sites, is intriguing. Twenty-five years of intein research has provided plentiful information on the nature of inteins, their distribution, mechanism of splicing, and use in biological applications. However, scientists must still look for possible intein-mediated regulation of essential genes and their role in microbial adaptation to stress conditions. Although recent studies have revealed a possible intein-mediated regulation of genes and their probable role in adaptation, further research is needed to understand and interpret their existence and distribution. The intein-mediated splicing mechanism (of making and breaking peptide bonds) has provided a tremendous tool to adapt in biological applications and also technological development. Nevertheless, there is no limit to the usability of the splicing mechanism in future applications.

## Figures and Tables

**Table 1:** Some of the intein-containing proteins involved in the DNA replication, recombination, and repair process.

Proteins	Function	Category	Organism
RecA/RadA	DNA-strand exchange	DNA repair	Eubacteria/Archaea
DnaB	Replicative DNA helicase	DNA replication	Eubacteria
Rad54/SWI-SNF2	dsDNA dependent ATPase	SF2 helicases & Chromatin remodeler	Eubacteria
UvrD/Rep/PcrA	ATP dependent DNA helicase (SF1 family)	DNA mismatch repair	Eubacteria
DnaE, Catalytic α-subunit of DNA pol III	DNA polymerase	DNA Replication	Eubacteria
Bacterial DNA polymerase I (PolA)	DNA synthesis	DNA Replication	Eubacteria
Bacterial DNA polymerase II (PolB)	DNA synthesis	DNA Replication	Eubacteria
DNA polymerase III τ and γ	DNA synthesis	DNA Replication	Eubacteria
RuvB	APT dependent DNA helicase	Holliday junction branch migration	Eubacteria
Ribonucleoisde diphosphate reductase	Ribonucleotide diphosphate reductase	DNA synthesis	Eubacteria; Archaea
DNA gyrase subunit A & B	Stabilizing the DNA (DNA replication, resection)	Topoisomerase	Eubacteria
DnaG	DNA primase	DNA replication	Eubacteria
RecG	ATP dependent DNA helicase	DNA replication	Eubacteria
Replication factor-C small unit (RFC)	DNA clamp loader	DNA Replication	Archaea
DNA polymerase II large unit (PolC/DP2)	DNA synthesis	DNA Replication	Archaea
DNA polymerase II small unit (PolB)	DNA synthesis	DNA Replication	Archaea
Mini-chromosome maintenance protein (MCM)	Replicative DNA helicase	DNA replication	Archaea

## Abbreviations

HED	Homing-endonuclease domain
3Rs	replication, repair and recombination
BIL	bacterial intein-like
SRR	sterol recognition region
DTT	dithiothreitol
NTPase	nucleotide tri-phosphotases
β-ME	beta-Mercaptoethanol
NCL	native chemical ligation
EPL	expressed protein ligation
PTS	protein trans-synthesis
CDB	chitin-binding domain
